# An Operation in the Park Bench Position Complicated by Massive Tongue Swelling

**DOI:** 10.1155/2012/165860

**Published:** 2012-03-05

**Authors:** Hiroyuki Koizumi, Satoshi Utsuki, Madoka Inukai, Hidehiro Oka, Shigeyuki Osawa, Kiyotaka Fujii

**Affiliations:** Department of Neurosurgery, Kitasato University School of Medicine, 1-15-1 Kitasato, Minami-ku, Sagamihara, Kanagawa 252-0375, Japan

## Abstract

This paper presents a case of massive tongue swelling as a complication after an operation in the park bench position. A 43-year-old male who had undergone a resection of a mass in the petrous bone of the clivus showed massive tongue swelling after the surgery in the left park bench position. A direct compression of the bite block caused the swelling of tongue. Tongue swelling may become fatal if it progresses to an airway obstruction; therefore the intraoperative and postoperative management is important.

## 1. Introduction

Intraoperative or postoperative complications of surgeries performed in the park bench position, such as bedsores, paralysis of the brachial plexus, and cervical cord injury, have been previously reported. This paper presents a case of massive tongue swelling which was a rare postoperative complication after the extirpation of a mass lesion in the petrous bone of the clivus which was performed in the park bench position. 

## 2. Case Report

**Figure 1:**
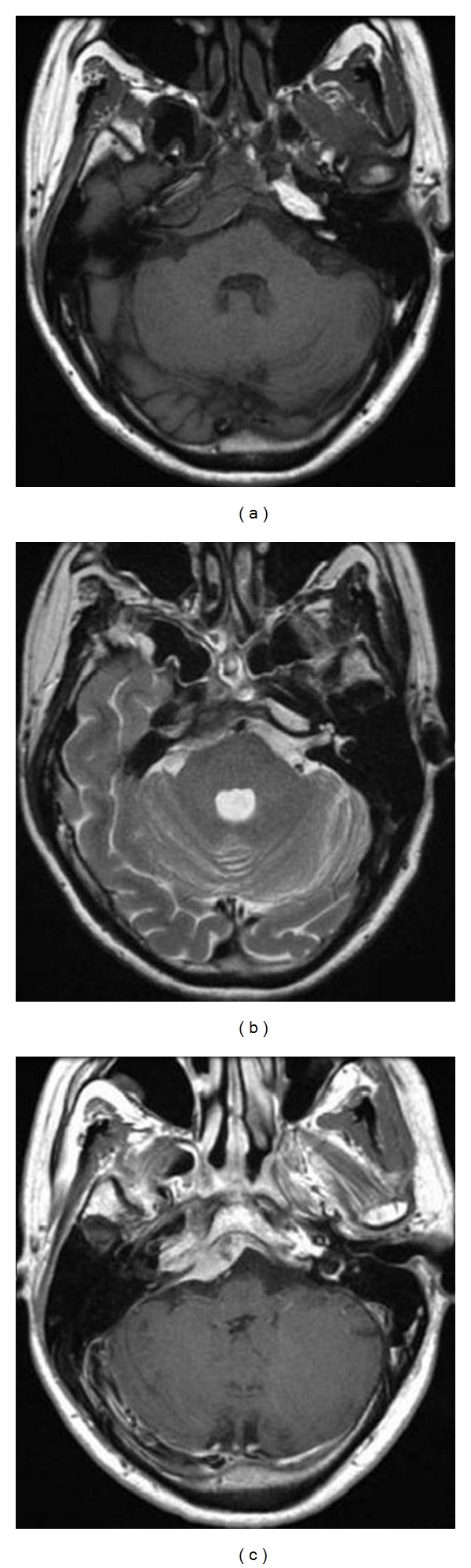
(a) Cerebral T1-weighted MRI shows a homogeneous, low-signal mass in the petrous bone from the right clivus. (b) Cerebral T2-weighted MRI reveals a homogeneous, high-signal mass in the petrous bone from the right clivus. (c) Cerebral T1-weighted MRI with gadolinium shows a heterogeneously enhanced mass in the petrous bone from the right clivus.

A 43-year-old male experienced a dull pain at the right back of his head. The headache thereafter evolved into right orbital pain. The patient subsequently developed gradually progressive diplopia. Cerebral magnetic resonance imaging (MRI) showed a mass lesion in the right clivus that was suspected to be a meningioma. The patient was admitted for surgery. A neurological examination showed no abnormalities other than right abducent nerve paralysis. Cerebral computed tomography (CT) revealed a tumor with homogenous enhancement which came in contact with the petrous bone from the right clivus. However, no osseous proliferative change was observed. Cerebral MRI revealed a mass appearing as homogeneously hypointense on T1, and hyperintense on T2-weighted images. T1-weighted MRI with gadolinium showed a heterogeneously enhanced mass in the petrous bone from the right clivus (Figure 1).

The patient underwent a resection of the mass via a left suboccipital craniotomy. Oral intubation was done with I.D 7.5 spiral tube using the Macintosh laryngoscope blade. Intubation was easy. The endotracheal tube was secured at a depth of 23 cm with a bite block and adhesive tape. The patient was then placed in the left park bench position, and the head was fixed with neck flexion. There was no excessive cervical flexion. Surgery was performed via the left lateral suboccipital approach. The lesion was not clearly visible, so hypertrophic dura mater was approved. There was no apparent tumor component, and the intraoperative pathological diagnosis was only fibrous tissue. The tissue was decompressed, and the operation was completed.

**Figure 2:**
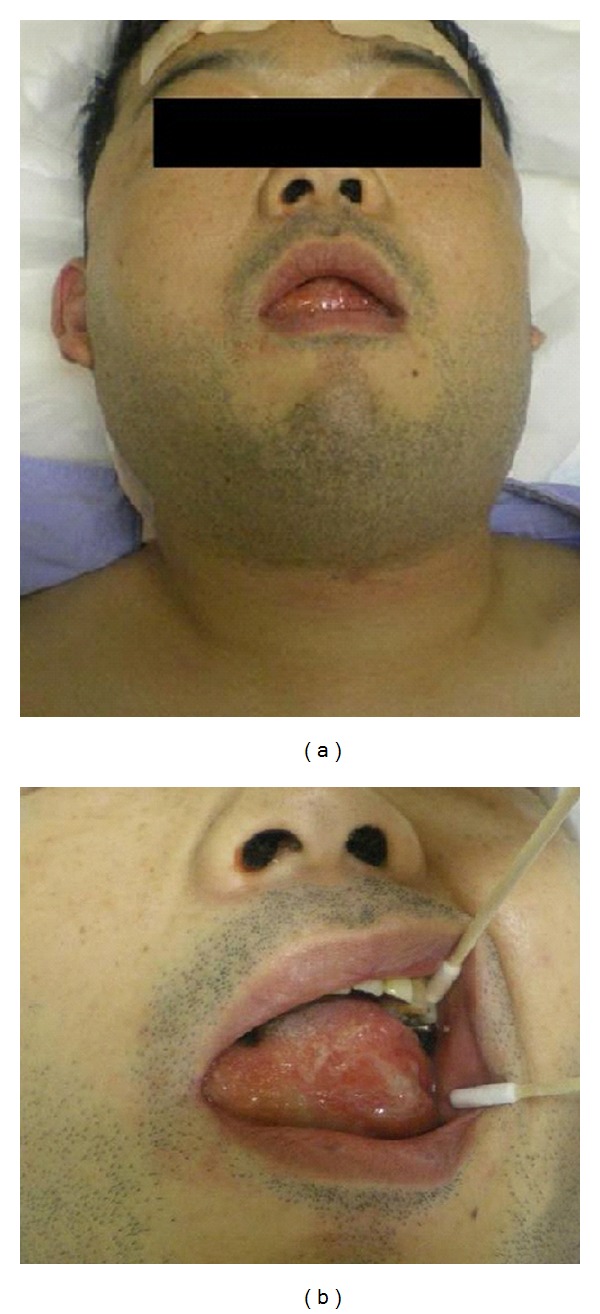
(a) Photograph showing neck and face swelling 13 hours after the operation. (b) Photograph showing tongue swelling on the left side 13 hours after surgery.

**Figure 3:**
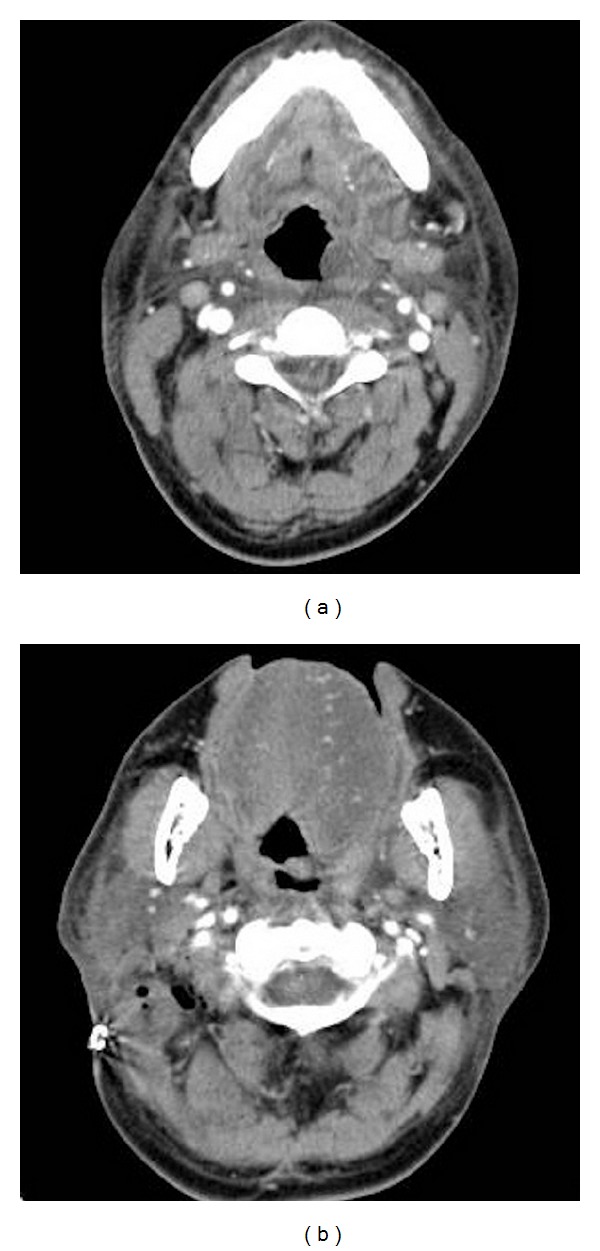
Postoperative contrast-enhanced computed tomography of the neck. Sixteen hours after the operation, swelling of the neck, tongue, pharynx, and vocal cords was observed on the left side.

**Figure 4:**
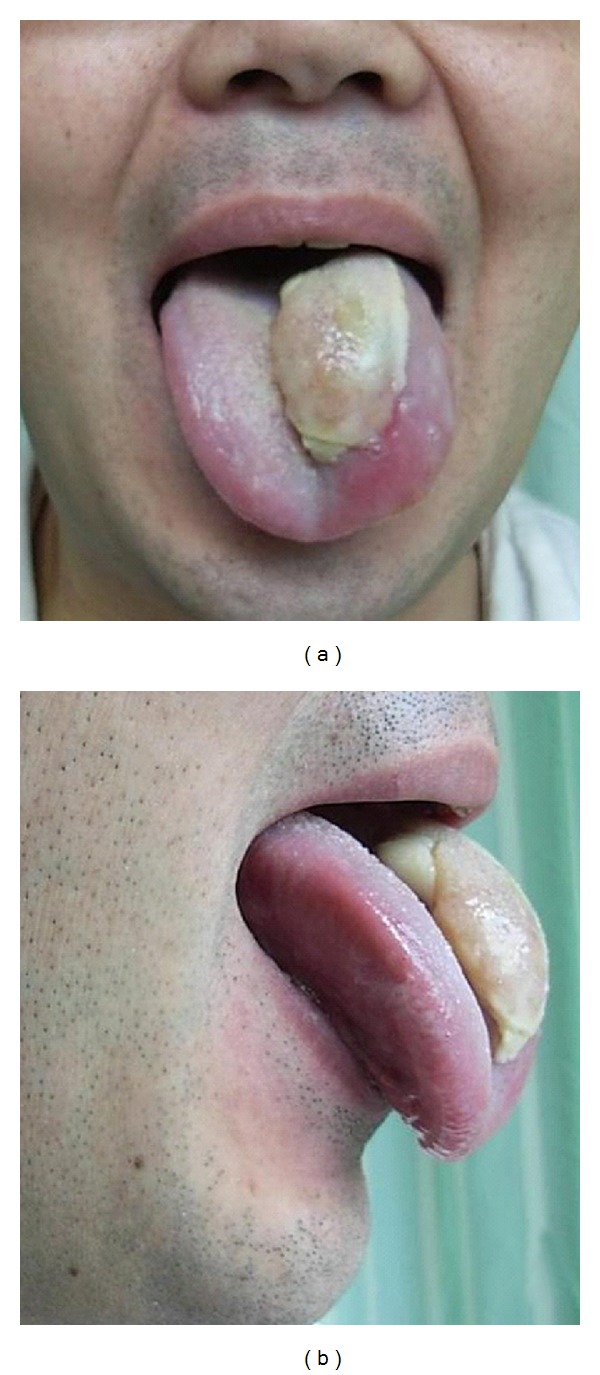
At 7 days after the operation, a photograph showing tongue swelling on the left side because of compression by the bite block.

The patient experienced pain in the tip of his tongue end upon returning to the intensive care unit. The tip of the tongue end on the left side showed a small ulcer. Mild neck swelling was observed 13 hours after the operation, but the swelling of the tongue was mild, and no airway narrowing was noted 13 hours after the operation (Figure 2). Neck CT 16 hours after the operation showed swelling of the neck, tongue, pharynx, and vocal cords on the left side (Figure 3). The ulcer became gradually larger on the left side of the tongue surface (Figure 4). The swelling of tongue gradually improved after the intravenous administration of steroids and the patient was discharged ten days later.

## 3. Discussion

**Figure 5:**
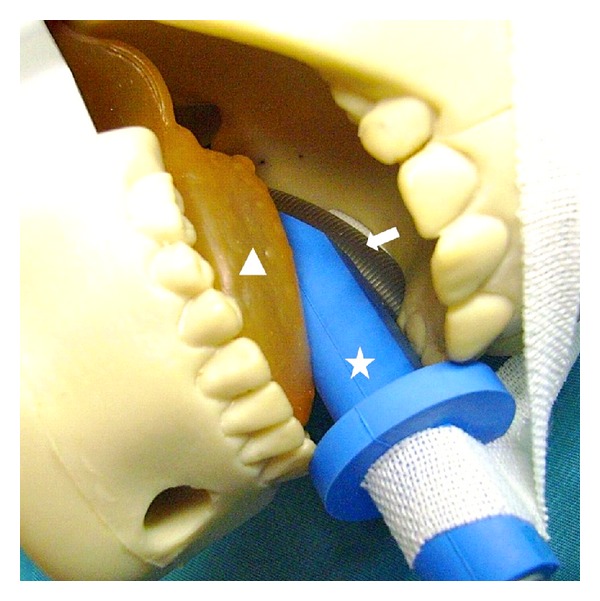
The photograph shows an intraoperative intraoral simulation model. The tongue (triangle) is injured by compression due to a bite block (star) and an intubation tube (arrow).

Tongue swelling is a rare a postoperative complication; however, it can be severe when it leads to a fatal upper airway obstruction. There have been several reported cases of tongue swelling due to venous congestion caused by the surgical position [1, 2], local mechanical compression of the tongue [3, 4], sublingual hematoma secondary to a difficult laryngoscopy and endotracheal intubation [5], and angioedema due to angiotensin converting enzyme inhibitor or Droperidol [6, 7]. Endotracheal intubation was easy in the current case, and neither droperidol nor angiotensin converting enzyme inhibitors were required. Therefore, the massive tongue swelling was not due to an injury at intubation or medicine-associated angioedema. Massive swelling of the neck and face was previously observed in a patient who underwent posterior fossa surgery in the park bench position; hence sufficient care was taken when utilizing this surgical position to minimize jugular compression by cervical flexion [8]. So far few venous return disorders have been reported in association with this operative position; therefore it was not thought to be the main cause of the excessive swelling of the patient's tongue. The cause in the present case was probably associated with an ulcer on the surface of the tongue surface that was associated with edema in the tongue and the surrounding tissue. In addition, reperfusion by compression discharge of a bite block caused tissue destruction and reflux disorder which occurred because the bite block had compressed the left side of the tongue for an extended period of time (Figure 5). The veins of the tongue are the dorsal lingual veins, which accompany the lingual artery; the deep lingual veins, which begin at the apex of the tongue, run posteriorly beside the lingual frenulum to join the sublingual vein. All these lingual veins terminate, either directly or indirectly, in the internal jugular vein. Venous return disorder may thus easily occur, thus resulting in the tongue becoming coecum when the base of tongue part is pressed (Figure 5). Due to the particular anatomy of the lingual vein, we must therefore pay attention to prevent the compression of an internal jugular vein by cervical flexion or the local compression of a lingual vein by a bite block and an intubation tube.

This patient showed swelling in his tongue, which did not progress to airway obstruction. Therefore, based on consultations with anesthesiology and otolaryngology personnel, prophylactic reintubation was not performed; however, his respiratory function was closely monitored due to the risk of airway obstruction. However, previous reports describe the use of reintubation following tongue swelling due to the occurrence of airway obstruction [9, 10]. Abe et al. noted that the airway should be established immediately by tracheal intubation with tongue swelling because the swelling often progresses rapidly, thus leading to airway obstruction [9].When massive tongue swelling occurs, the patient must be carefully monitored by pulse oximetry or ECG, and, depending on the severity of the edema, the supportive administration of steroids, antihistamines, and oxygen may thus be indicated. This is a point for discussion, since no evidence-based study has previously suggested this treatment to have any influence on edema [7, 11]. Therefore, it is evident that the most important treatment for such cases is to insure a free airway [7]. Due to the risk of fatal airway obstruction associated with swelling of the tongue, careful respiratory monitoring is required following extubation.

The localization of the bite block and intubation tube must be carefully evaluated during surgery to avoid congestion of the face and tongue. Swelling of the tongue after neurosurgical procedures has rarely been reported in the field of anesthesiology. Therefore, neurosurgeons must recognize how such a complication can occur and cooperate closely with anesthesiologists to appropriately treat such cases.
